# Clinical Features of Headache in Patients With Diagnosis of Definite Vestibular Migraine: The VM-Phenotypes Projects

**DOI:** 10.3389/fneur.2018.00395

**Published:** 2018-06-05

**Authors:** Roberto Teggi, Bruno Colombo, Roberto Albera, Giacinto Asprella Libonati, Cristiano Balzanelli, Angel Batuecas Caletrio, Augusto P. Casani, Juan Manuel Espinosa-Sanchez, Paolo Gamba, Jose A. Lopez-Escamez, Sergio Lucisano, Marco Mandalà, Giampiero Neri, Daniele Nuti, Rudi Pecci, Antonio Russo, Eduardo Martin-Sanz, Ricardo Sanz, Gioacchino Tedeschi, Paola Torelli, Paolo Vannucchi, Giancarlo Comi, Mario Bussi

**Affiliations:** ^1^ENT Department, San Raffaele Scientific Hospital, Milan, Italy; ^2^Headache Unit, Department of Neurology, San Raffaele Scientific Hospital, Milan, Italy; ^3^Dipartimento di Scienze, Chirurgiche Università di Torino, Turin, Italy; ^4^U. O. S. D. “Vestibologia e Otorinolaringoiatria” Presidio Ospedaliero “Giovanni Paolo II”, Policoro, Italy; ^5^Department of Otolaryngology, Spedali Civili, University of Brescia, Brescia, Italy; ^6^Otoneurology Unit, Department of Otorhinolaryngology, University Hospital of Salamanca, IBSAL, Salamanca, Spain; ^7^Skull Base Unit, Department of Otorhinolaryngology, University Hospital of Salamanca, IBSAL, Salamanca, Spain; ^8^Department of Otorhinolaryngology, Pisa University Medical School Otorhinolaryngology, Pisa University Medical School, Pisa, Italy; ^9^Otology and Neurotology Group CTS495, Department of Genomic Medicine- Centro de Genómica e Investigación Oncológica - Pfizer/Universidad de Granada/Junta de Andalucía (GENYO), Granada, Spain; ^10^Division of Otoneurology, Department of Otolaryngology, Instituto de Investigación Biosanitaria ibs.GRANADA, Hospital Virgen de las Nieves, Granada, Spain; ^11^Department of Otorhinolaryngology—Head and Neck Surgery, Poliambulanza Foundation Hospital, Brescia, Italy; ^12^Otology and Skull Base Unit, Azienda Ospedaliera Universitaria Senese, Siena, Italy; ^13^Department of Neurosciences, Imaging and Clinical Sciences, University of Chieti-Pescara, Chieti, Italy; ^14^Unit of Audiology, Department of Surgical Sciences and Translational Medicine, Careggi Hospital, University of Florence, Florence, Italy; ^15^Department of Otolaryngology, University Hospital of Getafe, Madrid, Spain; ^16^Department of Neurology, University of Campania Luigi Vanvitelli, Naples, Italy; ^17^Department of Neurosciences, Headache Centre, University of Parma, Parma, Italy

**Keywords:** Vestibular Migraine, vertigo, headache, migraine, clinical diagnosis, vestibular disorders

## Abstract

Migraine is a common neurological disorder characterized by episodic headaches with specific features, presenting familial aggregation. Migraine is associated with episodic vertigo, named Vestibular Migraine (VM) whose diagnosis mainly rely on clinical history showing a temporary association of symptoms. Some patient refers symptoms occurring in pediatric age, defined “episodic symptoms which may be associated with migraine.” The aim of this cross sectional observational study was to assess migraine-related clinical features in VM subjects. For the purpose, 279 patients were recruited in different centers in Europe; data were collected by a senior neurologist or ENT specialist through a structured questionnaire. The age of onset of migraine was 21.8 ± 9. The duration of headaches was lower than 24 h in 79.1% of cases. Symptoms accompanying migrainous headaches were, in order of frequency, nausea (79.9%), phonophobia (54.5%), photophobia (53.8%), vomiting (29%), lightheadedness (21.1%). Visual or other auras were reported by 25.4% of subjects. A familial aggregation was referred by 67.4%, while migraine precursors were reported by 52.3% of subjects. Patients reporting nausea and vomiting during headaches more frequently experienced the same symptoms during vertigo. Comparing our results in VM subjects with previously published papers in migraine sufferers, our patients presented a lower duration of headaches and a higher rate of familial aggregation; moreover some common characters were observed in headache and vertigo attacks for accompanying symptoms like nausea and vomiting and clustering of attacks.

## Introduction

Migraine is one of the most prevalent neurological disorder with an estimated number of more than 80 millions sufferers in US and Europe (1, 2). According to the International Headache Society (HIS) criteria, typically the migrainous headaches last between 4 and 72 h and present at least two of the following features:

- Unilateral.
- Pulsating.
- Moderate or severe intensity of pain.
- Aggravated by, or resulting in the avoidance of, routine physical activity.

In addition, there is at least one of:

- Nausea and vomiting during migraine attacks.
- Photophobia and phonophobia.

Visual and other aura may precede the headache (3, 4).

In clinical practice the association of vertigo and migraine is of common observation. Particularly, the complaint of vertigo is more elevated in migraineurs than in general population, ranging from 30 to 50% (5, 6). In previous years, various terms such as migraine-associated dizziness, migraine-related vertigo, and migrainous vertigo have been used to describe episodic vestibular symptoms in patients with migraine when an alternative diagnosis has been ruled out (7, 8). Since clinical examination is usually normal during the vertigo-free period (9) the diagnosis mainly relies on clinical history. Recently, a joint committee between the International Headache Society and the Bárány Society established the following diagnostic criteria for both definite and probable vestibular migraine (VM), the nosological disorder in which migraine is associated with the occurrence of vestibular symptoms and other causal factors are ruled out (10).

Definite VM:

- At least five episodes with vestibular symptoms of a moderate or severe intensity, lasting 5 min to 72 h.
- Current or previous history of migraine with or without aura according to the International Classification of Headache Disorders (ICHD).
- One or more migraine features with at least 50% of the vestibular episodes:
  - Headache with at least two of the following characteristics: one-sided location, pulsating quality, moderate or severe pain intensity, aggravation by routine physical activity
  - Photophobia and phonophobia
  - Visual aura
- Not better accounted for by another vestibular or ICHD diagnosis

In order to diagnose probable VM, only one of the criteria B or C must be observed.

The diagnostic criteria for definite VM are included in the 3rd edition of the International Classification of Headache Disorders (ICHD-III), published in 2013, where it appears in the appendix for new disorders (3).

Familial aggregation has been widely observed in migraine (11) and episodic vertigo (12), and both disorders may arise from the interplay between genetic predisposition and environmental factors.

Finally, ICHD-III included four migraine equivalents in infancy and childhood, defined as “episodic syndromes which may be associated with migraine”: cyclic vomiting, abdominal pain, benign paroxysmal vertigo, and benign paroxysmal torticollis. For some of them, an involvement of the vestibular system can be proposed. Motion sickness in pediatric subjects, consisting of autonomic signs and symptoms occurring during movement, has also been studied as a possible migraine precursor (13).

The aim of our work was to assess the features of headaches, accompanying symptoms, familial history and migraine precursors in a large sample of subjects with a diagnosis of definite VM and the relationship with accompanying symptoms of vertigo episodes.

## Materials and methods

### Patients

In this cross-sectional observational multicentric study, 279 subjects were recruited in different centers in Italy and Spain between January 2016 and March 2017. Participating centers (in order of number of enrolled patients) were Naples (51 patients), Milan (45), Brescia (45), Florence (23), Turin (20), Granada (19), Pisa (16), Parma (15), Salamanca (12), Matera (12), Siena (10), Madrid (6), and Chieti (5). All patients presented a diagnosis of definite VM according to the Bárány/ICHD Society criteria (10). To be included, all patients should have at least five attacks of headache with migrainous features. Of the 279 subjects, 235 (84.2%) were female. The age at inclusion was 45.8 ± 13.8 years (range 18–78 years). All patients were interviewed by a senior neuro-otologist. A six months' follow-up was required to confirm the diagnoses before inclusion. The vestibular phenotype of 252 of the 279 subjects included in this study was previously published (14).

Six patients were excluded after the follow up since they developed a low-frequencies sensorineural hearing loss possible related to hydrops. The centers were tertiary referral outpatient clinics or vertigo clinics in general hospitals. This study was approved by the local ethics committees in all of the participating centers. Data were saved on eCRF in our hospital, only senior specialists were given username/password and they were asked to include patients evaluated in their clinical activity presenting criteria for definite VM and signing informed consent.

### Methods

A full neurotological examination including video HIT, video-nystagmography with caloric testing, an audiometric examination and a CNS MRI with contrast was performed before inclusion. Patients with low frequency sensorineural hearing loss were not included. A diagnosis of migraine was confirmed by a senior neurologist. A structured questionnaire was designed to record the headache characters and accompanying symptoms.

The questionnaire also included patient's age at onset of vestibular symptoms and headache, and a set of questions to determine the accompanying symptoms occurring during the attacks of headache (nausea, vomiting, phono\photophobia, blurred or double vision, feeling lightheaded, weakness of arms and legs, visual aura). Nausea and vomiting occurring during vestibular episodes were also saved. Accompanying symptoms were considered only when occurring in at least 50% of attacks.

Patients were also asked if any of their family members, i.e., another relative in the first or second degree, suffered from migraine in the family.

Finally, the following migraine precursors were investigated in the questionnaire: motion sickness, cyclic vomiting, episodic abdominal pain, episodic vertigo, torticollis (13). To diagnose migraine precursors, criteria of ICHD III beta version were observed (3).

A positive history for cyclic vomiting has been considered when the patient referred episodes (at least 5) of severe vomiting without apparent cause, lasting for at least 1 h or days alternating with symptom-free periods and occurring with the same stereotyped symptoms and intensity in childhood. Episodic abdominal pain was considered when patients reported recurrent attacks of moderate to severe midline abdominal pain, associated with vasomotor symptoms, nausea and vomiting, lasting 2–72 h and with normality between episodes. Torticollis was diagnosed when the patient reported recurrent and stereotyped episodes of head tilt to one side, perhaps with slight rotation, which remit spontaneously occurring in infants and small children, with onset in the first year. BPV was diagnosed in subjects reporting recurrent brief attacks of vertigo, occurring without warning and resolving spontaneously, in otherwise healthy children.

We included motion sickness among them, although not commonly accepted, and probably better definable migraine marker.

### Statistical analysis

Absolute and relative frequencies of each symptom were calculated and compared between different groups using Chi-squared statistics. Quantitative variables are presented as mean ± standard deviation and unpaired Student's *t-*tests were used to compare them. A *p* < 0.05 was considered to be statistically significant. A Spearman test was performed to investigate the association between different variables and a Bonferroni correction was applied. One full regression model was calculated to assess the independent role of migraine precursors on age of onset of vertigo. We used SPSS software version 17.0 (SPSS, Inc., Chicago, IL, USA) for statistical analysis.

## Results

In our sample the first headache occurred at 21.8 ± 9 (ranging between 12 and 50 years old), while the first vertigo at 37.4 ± 13.1 (ranging between 17 and 70). In 19 cases patients reported synchronous occurrence of headache and vertigo; these patients presented a lower age of onset of both disorders (mean age of 19.8 ± 2.1, *p* < 0.05). The female\male ratio was 5.3/1.

The number of attacks of headache in the last 12 months was 1.09 (ranging between 0.5 and 2).

In Table 1 are reported the localization of the headaches in our sample, while Table 2 reports the duration of headaches in the sample; no difference was detected for the age of onset of headaches in subgroups with different duration of headaches. Investigators were asked to save the most frequent location and duration of headaches.

**Table 1:** Localization of headaches; in the second column the total number of subjects (and percentages).

**Localization of headaches**	**Total number (and percentages)**
Above right or left eyebrow	26 (9.3%)
Above both eyebrows	24 (8.6%)
Behind right or left eye	26 (9.3%)
Behind both eyes	24 (8.6%)
Right or left temporal region	48 (17.2%)
Both temporal regions	68 (24.4%)
Back of the head on right or left	27 (9.7%)
Back of the head on both sides	36 (12.9%)

*Most frequent locations have been saved*.

**Table 2:** Duration of headache attacks; in the second column the total number of subjects (and percentages).

**Duration of headache**	**Total number (and percentage)**
Within 2 h	17 (6.2%)
3–4 h	43 (15.5%)
5–12 h	55 (19.8%)
12–24 h	104 (37.6%)
1–3 days	58 (20.8%)
More than 1 week	2 (0.1%)

*The most frequent duration was saved; patients were included if they had at least 5 headaches lasting more than 4 h*.

Accompanying symptoms occurring during headaches are reported in Table 3.

**Table 3:** Accompanying symptoms of headaches; in the second column the total number of subjects (and percentages) referring symptom in at least 50% of attacks.

**Accompanying symptoms**	**Total number (and percentage)**
Phonophobia	152 (54.5%)
Photophobia	150 (53.8%)
Lightheadedness	59 (21.1%)
Blurred\Double vision	22 (7.9%)
Loss of vision	17 (6.1%)
Weakness of arms and legs	23 (9.5%)
Nausea	223 (79.9%)
Vomiting	81 (29%)

A correlation has been found between nausea and vomiting during headaches and during vertigo; in the subgroup of 223 patients referring nausea in at least 50% of headaches, 145 (65%) also referred nausea during vertigo, while 27 (48%) of 56 subjects without nausea during headaches (χ^2^ = 5.35, *p* = 0.02). Similarly, in the subgroup of 81 patients referring vomiting during headaches, 46 (56.8%) also referred vomiting during vertigo, while 8 (4%) in the subgroup of 198 subjects with a negative history of vomiting during headaches reported vomiting during vertigo (χ^2^ = 107, *p* ≤ 0.0001).

Seventy-one (25.4%) subjects referred visual or other auras preceding headaches; they did not present differences compared with the total sample for the age of onset of headache (21.2 ± 8.3).

One hundred-eighty eight (67.4%) subjects reported familial cases of headaches with migrainous features among relatives of first or second degree. Among 71 subjects with aura, 62 (87.3%) reported familial cases of migraine (*p* = 0.001).

One hundred forty-six subjects (52.3%) reported a positive history for at least one of migraine precursors. The total number and frequency are reported in the Table 4.

**Table 4:** Migraine precursors in VM patients; in the second column the total number (and percentage) of patients.

**Migraine precursors**	
Motion sickness	117 (41.9%)
Cyclic vomiting	24 (8.6%)
Episodic abdominal pain	18 (6.5%)
Episodic vertigo (BPV)	12 (4.3%)
Torticollis	12 (4.3%)

A Spearman test demonstrated a correlation between a positive history for at least one precursor and age of occurrence of the first headache (*t* = 2.31, *p* = 0.02). No statistics has been found between the presence of precursors and clinical features of headaches.

Finally, 40 patients (14.3%) reported headaches clustered in specific periods, while 54 for vertigo attacks (19.3%). A strong correlation has been found between the two variables (*t* = 9.75, *p* ≤ 0.0001).

## Discussion

Vestibular Migraine (VM) is a common variant of migraine, resulting in recurrent vestibular symptoms in association with migraine features. It is the most frequent central cause of episodic vertigo and the second most important cause of vertigo overall (15).VM diagnosis is primarily based on clinical history (16). Admittedly, symptoms may be heterogeneous among VM patients; in a previous paper we presented data regarding vestibular manifestations on a sample of subjects fulfilling VM criteria (14).

From this background, VM-Phenotypes project has the purpose to better elucidate the patients' characteristics and phenotypes by assessing a large sample of probands with definite VM diagnosis.

Data were collected during clinical activity in specialized Headache and ENT Departments.

The VM diagnosis rely on very accurate anamnestic self-report data, focused on migraine and vestibular symptoms: since the structured interview was performed by a Senior Neurologist or a Senior Otologist in tertiary University Clinics, we were able to consecutively include 279 patients with a confirmed diagnosis of definite VM.

In our Caucasian group of VM patients, 84.2% were females with an age at inclusion of 45.8 years. This VM female prevalence is higher in comparison with Caucasian female:male migraine (with or without aura) prevalence at the same age (3.5:1) (17).

The onset of migraine preceded the onset of vertigo by around 15 years, while the age of the first episode of migraine in VM patients does not demonstrate differences with that of general migraineurs population (2).

Interestingly, in VM patients reporting a synchronous onset of migraine and vertigo, the age of onset of both is lower. On the opposite, we found no difference for onset of headaches in VM patients with or without aura.

Considering the well-known aggregation of migraine within families, it could be speculated a genetic contribution in this group of VM patients.

Moreover, focusing on putative genetic aspects, we found a considerable high rate of VM patients with a familial history of migraine (67.4%) compared to previously published data on general population of migraineurs reporting an heritability ranging from 34 to 57% (18).

As far as migraine symptoms are concerned, in our VM patients' sample duration of migraine is variable, although a high rate of VM patients (79.1%) reported attacks lasting <24 h. This is in contrast with classical studies whereas among women, ~71% of attacks last longer than 24 h (19).

In our group of VM patients we found a high percentage (69%) of subjects referring at least one of the pediatric precursors of migraine, with a particular preminence of motion sickness (42.8%).

As far as the rate of migraine precursors in pediatric age is concerned, these data are in line with previous published works (20).

In our knowledge this is the first work describing a possible relationship between migraine precursors and onset of headache on adulthood. Considering data on migraine precursor, we can speculate about some possible predisposing factor in childhood to eventually develop VM.

As a final consideration, in VM subjects some “specific features” are maintained in vertigo and headache attacks; above all, patients reporting nausea and vomiting during headaches more easily experience nausea and vomiting during vertigo. Moreover, clustering of headaches and vertigo more easily happens in specific patients. It could be speculated that an “autonomic hypersensitivity” in these subjects may play a role in the finding.

In summary, in our group of Caucasian VM patients, preminent phenotypic migraine features are: female patients with migraine attacks of moderate to severe intensity, duration less of 24 h, low frequency (one attack per month) and predisposing factors such as familiarity and precursors in pediatric age (motion sickness in particular).

Finally, we want to underline some possible limitations of our study. Above all, evaluation of familial cases and clinical history of migraine precursors may present some uncertainty, since these data were collected mainly during the structured interview with the patient.

Admittedly, diagnosis of VM remains a challenge for Neurologists and ENT specialists.

The knowledge of disease course and phenotype characteristics from the pediatric age to adulthood could be essential to provide an early diagnosis, a reliable prognosis and a valuable care for VM patients.

## Ethics statement

This study was carried out in accordance with the recommendations of name of guidelines, name of committee with written informed consent from all subjects. All subjects gave written informed consent in accordance with the Declaration of Helsinki. The protocol was approved by the name of committee.

## Author contributions

RT and BC performed statistical analysis of data and wrote the paper. Data were collected by all other participants. All authors agree with the conclusions.

### Conflict of interest statement

The authors declare that the research was conducted in the absence of any commercial or financial relationships that could be construed as a potential conflict of interest.
