# Corrigendum: Clinical Features of Headache in Patients With Diagnosis of Definite Vestibular Migraine: The VM-Phenotypes Projects

**DOI:** 10.3389/fneur.2019.01374

**Published:** 2020-01-24

**Authors:** Roberto Teggi, Bruno Colombo, Roberto Albera, Giacinto Asprella Libonati, Cristiano Balzanelli, Angel Batuecas Caletrio, Augusto P. Casani, Juan Manuel Espinosa-Sanchez, Paolo Gamba, Jose A. Lopez-Escamez, Sergio Lucisano, Marco Mandalà, Giampiero Neri, Daniele Nuti, Rudi Pecci, Antonio Russo, Eduardo Martin-Sanz, Ricardo Sanz, Gioacchino Tedeschi, Paola Torelli, Paolo Vannucchi, Giancarlo Comi, Mario Bussi

**Affiliations:** ^1^ENT Department, San Raffaele Scientific Hospital, Milan, Italy; ^2^Headache Unit, Department of Neurology, San Raffaele Scientific Hospital, Milan, Italy; ^3^Dipartimento di Scienze, Chirurgiche Università di Torino, Turin, Italy; ^4^U. O. S. D. “Vestibologia e Otorinolaringoiatria” Presidio Ospedaliero “Giovanni Paolo II”, Policoro, Italy; ^5^Department of Otolaryngology, Spedali Civili, University of Brescia, Brescia, Italy; ^6^Otoneurology Unit, Department of Otorhinolaryngology, University Hospital of Salamanca, IBSAL, Salamanca, Spain; ^7^Skull Base Unit, Department of Otorhinolaryngology, University Hospital of Salamanca, IBSAL, Salamanca, Spain; ^8^Department of Otorhinolaryngology, Pisa University Medical School Otorhinolaryngology, Pisa University Medical School, Pisa, Italy; ^9^Otology and Neurotology Group CTS495, Department of Genomic Medicine- Centro de Genómica e Investigación Oncológica - Pfizer/Universidad de Granada/Junta de Andalucía (GENYO), Granada, Spain; ^10^Division of Otoneurology, Department of Otolaryngology, Instituto de Investigación Biosanitaria Ibs.GRANADA, Hospital Virgen de las Nieves, Granada, Spain; ^11^Department of Otorhinolaryngology—Head and Neck Surgery, Poliambulanza Foundation Hospital, Brescia, Italy; ^12^Otology and Skull Base Unit, Azienda Ospedaliera Universitaria Senese, Siena, Italy; ^13^Department of Neurosciences, Imaging and Clinical Sciences, University of Chieti-Pescara, Chieti, Italy; ^14^Unit of Audiology, Department of Surgical Sciences and Translational Medicine, Careggi Hospital, University of Florence, Florence, Italy; ^15^Department of Otolaryngology, University Hospital of Getafe, Madrid, Spain; ^16^Department of Neurology, University of Campania Luigi Vanvitelli, Naples, Italy; ^17^Department of Neurosciences, Headache Centre, University of Parma, Parma, Italy

**Keywords:** Vestibular Migraine, vertigo, headache, migraine, clinical diagnosis, vestibular disorders

In the original article, there was an error. The authors regret that, although it was mentioned in the **Discussion** section, it was not made sufficiently clear in the **Materials and Methods** section that the results presented on headache features are based, for the most part, on the same patients' population as described in a previous paper (see reference 16), in which however, only the vestibular aspects were reported.

A correction has been made to the **Materials and Methods** section, subsection **Patients, paragraph one:**

“In this cross-sectional observational multicentric study, 279 subjects were recruited in different centers in Italy and Spain between January 2016 and March 2017. Participating centers (in order of number of enrolled patients) were Naples (51 patients), Milan (45), Brescia (45), Florence (23), Turin (20), Granada (19), Pisa (16), Parma (15), Salamanca (12), Matera (12), Siena (10), Madrid (6), and Chieti (5). All patients presented a diagnosis of definite VM according to the Bárány/ICHD Society criteria (10). To be included, all patients should have at least five attacks of headache with migrainous features. Of the 279 subjects, 235 (84.2%) were female. The age at inclusion was 45.8 ± 13.8 years (range 18–78 years). All patients were interviewed by a senior neuro-otologist. A six months' follow-up was required to confirm the diagnoses before inclusion. The vestibular phenotype of 252 of the 279 subjects included in this study was previously published (14).”

The authors apologize for this error and state that this does not change the scientific conclusions of the article in any way. The original article has been updated.
